#  Psychoradiological investigations of gray matter alterations in patients with anorexia nervosa

**DOI:** 10.1038/s41398-018-0323-3

**Published:** 2018-12-13

**Authors:** Simin Zhang, Weina Wang, Xiaorui Su, Graham J. Kemp, Xibiao Yang, Jingkai Su, Qiaoyue Tan, Youjin Zhao, Huaiqiang Sun, Qiang Yue, Qiyong Gong

**Affiliations:** 1 Huaxi MR Research Center (HMRRC), Department of Radiology, West China Hospital of Sichuan University, 610041 Chengdu, China; 2 Liverpool Magnetic Resonance Imaging Centre (LiMRIC) and Institute of Ageing and Chronic Disease, University of Liverpool, Liverpool, UK; 3 Department of Radiology, West China Hospital of Sichuan University, No. 37 Guo Xue Xiang, 610041 Chengdu, China

## Abstract

Anorexia nervosa (AN) is a severe psychiatric disorder with high mortality. The underlying neurobiological mechanisms are not well understood, and high-resolution structural magnetic resonance brain imaging studies have given inconsistent results. Here we aimed to psychoradiologically define the most prominent and replicable abnormalities of gray matter volume (GMV) in AN patients, and to examine their relationship to demographics and clinical characteristics, by means of a new coordinate-based meta-analytic technique called seed-based d mapping (SDM). In a pooled analysis of all AN patients we identified decreased GMV in the bilateral median cingulate cortices and posterior cingulate cortices extending to the bilateral precuneus, and the supplementary motor area. In subgroup analysis we found an additional decreased GMV in the right fusiform in adult AN, and a decreased GMV in the left amygdala and left anterior cingulate cortex in AN patients without comorbidity (pure AN). Thus, the most consistent GMV alterations in AN patients are in the default mode network and the sensorimotor network. These psychoradiological findings of the brain abnormalities might underpin the neuropathophysiology in AN.

## Introduction

Anorexia nervosa (AN) is a serious and distinctive psychiatric disorder, particularly affecting adolescent girls and young adult women^1^. Although relatively rare (prevalence ~0.3%), AN has serious medical consequences (mortality ~10%) and thus poses a major clinical, psychological, and societal burden^2^. As defined in the fifth revision of the Diagnostic and Statistical Manual of Mental Disorders (DSM-5; American Psychiatric Association, 2013), AN is characterized by an intense fear of weight gain and a distorted view of body shape, which motivates severe dietary restriction or other weight loss behaviors, such as purging or excessive physical activity^3^. Cognitive and emotional functioning are markedly disturbed, and serious medical morbidity and psychiatric comorbidity are common^4^. AN has a complex etiology, involving genetic/neurobiological, social–environmental and psychological factors^5^.

Radiological techniques such as magnetic resonance imaging (MRI) is an important psychoradiologic technique (https://radiopaedia.org/articles/psychoradiology)^64–67^. In AN, a number of MRI studies have employed the analytical technique of voxel-based morphometry (VBM); this avoids some limitations of region-of-interest (ROI) approaches, which focus on selected regions and preclude the exploration of other brain regions that may be involved.

A significant global loss of brain volume in AN, regarding both gray matter (GM) and white matter (WM), has been identified in several studies^6–9^. However, studies investigating regional changes in AN have yielded less consistent results, reporting reduced volumes in a wide variety of areas including cingulate cortex (anterior cingulate cortices (ACC), median cingulate cortices (MCC), posterior cingulate cortices (PCC)), frontal lobe (supplementary motor area (SMA), inferior frontal gyrus (IFG), and frontal operculum), temporal lobe (the superior/middle temporal gyrus (STG/MTG), fusiform, and temporoparietal junction), parietal lobe (precuneus and inferior parietal cortex), occipital cortex, cerebellum, and striatum^6,7,10–23^. While one recent study found increased gray matter volume (GMV) in the left orbitofrontal gyrus rectus, bilateral fusiform gyrus, bilateral hippocampus, right insula, and bilateral parahippocampal gyrus^24^, another found no significantly reduced GMV in the hypothalamus^25^. Taken together, these studies have not identified any common theme with respect to functionally important regions that throw light on the neurobiological factors underlying AN.

Meta-analysis is a powerful tool which integrates multiple studies of a particular problem to derive insights often unavailable from the studies individually. To our knowledge only one meta-analysis has compared GMV differences between AN patients and healthy control (HC) subjects using the method of activation likelihood estimation (ALE)^8^. This implicated reward and somatosensory abnormalities in AN, reporting decreased GMV in hypothalamus, striatum (caudate nucleus, lentiform nucleus) and the inferior parietal lobe, with no significant GMV increases. However, the number of published primary VBM studies in AN at that time was small, only seven being included; furthermore, the meta-analysis did not consider confounding factors, such as differences in age, psychiatric, and medical comorbidity, and duration of illness^8^. Now a further 14 primary AN VBM studies have been published, it is timely to conduct an updated meta-analysis to help define GMV alterations in AN.

The aims of this paper were threefold. First, we performed pooled meta-analyses of all included studies to identify consistent GMV changes in AN. Second, we conducted subgroup meta-analyses to assess the robustness and heterogeneity of the main findings. Finally, we used meta-regression methods to examine the effects of demographics and clinical characteristics. We hypothesized that AN patients would show reduced GMV in some functionally important regions, such as cingulate cortex and striatum which may help to account for the symptomatology. We also hypothesized that the two subgroups of adult AN and AN without comorbidity would show distinctive GMV abnormalities.

## Methods

### Study selection

A systematic strategy was used to search for relevant studies published in PubMed, Embase, Web of Science, and Google Scholar up to May 2018 using combinations of the terms “anorexia nervosa” or “AN” or “eating disorder” plus “VBM” or “voxel-based morphometry” or “whole brain” or “morphometric”. The reference lists of these studies were manually checked to identify additional studies.

The following were criteria for inclusion: (i) an original article in a peer-reviewed journal; (ii) including patients with a primary diagnosis of AN based on DSM criteria; (iii) reporting a VBM case-control study on AN patients and HC subjects; (iv) reporting whole-brain GMV alterations in a stereotactic space in three-dimensional standard coordinates; (v) using significance thresholds for data that were either corrected for multiple comparisons or uncorrected with spatial extent thresholds. If necessary, corresponding authors were contacted by e-mail to provide details not in the original manuscripts. Studies were excluded if: (i) it was impossible to obtain the three-dimensional coordinates in stereotactic space; (ii) the data overlapped with those of other publications (if so, the study with the larger sample size was selected); (iii) there was no HC group; (iv) only region of interest (ROI) findings were reported; (v) the findings were based on small-volume correction; (iv) studies reported recovered AN patients. We followed the preferred reporting items for systematic reviews and meta-analysis (PRISMA) guidelines^26^.

Three authors (Z.S.M., W.W.N., and S.X.R.) independently searched the literature, examined the retrieved articles, extracted and cross-checked data. The results were compared, and any inconsistencies were resolved by consensus. The coordinates in each study were extracted for meta-analysis according to the SDM method.

### Voxel-wise meta-analysis by SDM

The analytical processes are described in the SDM tutorial (http://sdmproject.com/sofware/Tutorial.pdf) and related publications. SDM has been widely applied to e.g. childhood maltreatment^27^, obsessive-compulsive disorder (OCD)^28^, and major depressive disorder (MDD)^29^. The approach creates effect size and variance maps based on reported peak coordinates, which are then analyzed with traditional random-effects meta-analytic methods. In addition, this technique allows heterogeneity maps to be generated and meta-regressions to be conducted across the whole brain. Importantly, SDM also allows meta-analytic group comparisons, which provide an indication of whether computed effect sizes differ significantly between groups^30,31^. In the current version of SDM^31^, a standard Montreal Neurological Institute (MNI) map of the differences in GMV was separately recreated for each included study using an anisotropic Gaussian kernel, which assigns higher effect sizes to the voxels that are more correlated with peaks. These anisotropic kernels optimize the recreation of the effect size maps and provide greater robustness, because they do not depend on a full width at half maximum (FWHM).

We planned to conduct pooled meta-analysis of all the included studies, and then four subgroup analyses: adult AN patients; adolescent AN patients; AN patients with comorbidity; and AN patients without comorbidity (‘pure AN’). However, there were too few studies to allow subgroup analyses of adolescents and AN with comorbidity (a minimum of 10 studies is recommended for SDM meta-analyses^32^). To ensure that only the most replicable and robust of the results were retained, a jackknife sensitivity analysis was conducted. The meta-regression analyses were conducted with relevant clinical variables, including BMI, age, illness duration, percentage of females, and percentage of medicated patients as regressors.

A threshold of *p* < 0.005 with peak *Z* > 1 and a cluster extent of >10 voxels was used for the meta-analyses and heterogeneity analyses^30^.

## Results

### Included studies and sample characteristics

**Fig. 1:**
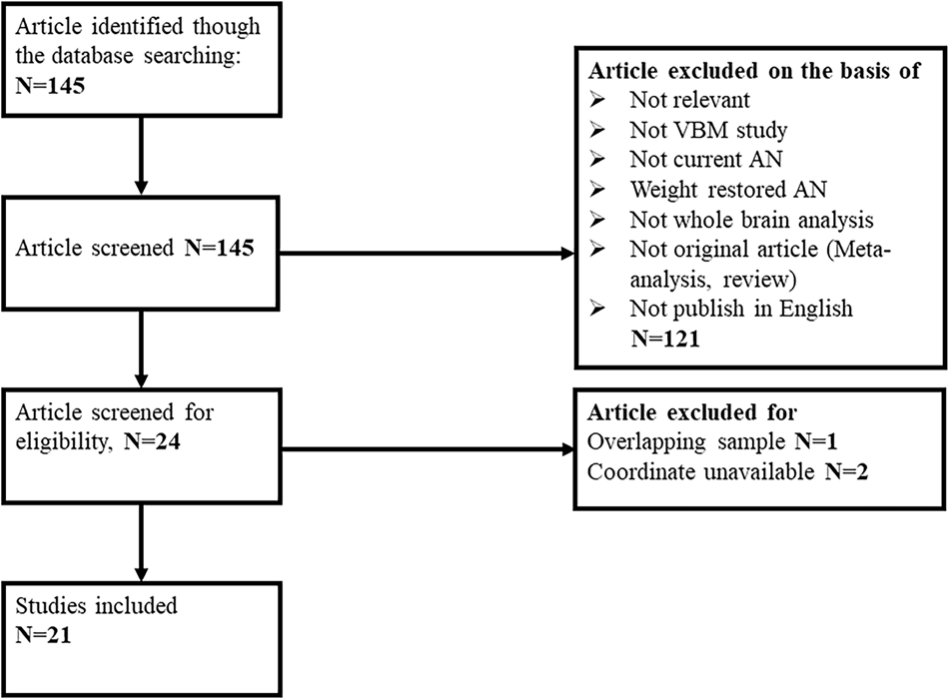
Search strategy used for the inclusion of the studies considered in the current meta-analysis. AN anorexia nervosa, VBM voxel-based morphometry

The search strategy initially identified 145 studies, of which 21 studies met the inclusion criteria (summarized in Fig. 1). Our final sample comprised 389 AN patients and 410 HC. Table 1 summarizes clinical and demographic data from all included studies. Table 2 summarizes technique details from all included studies. In no study was there any significant difference in age and sex between AN and HC groups.

**Table 1:** Demographic and clinical characteristics of subjects in the 21 voxel-based morphometry data sets included in the meta-analysis

Study	Number (female)		Age (years)		Duration (years)	BMI		BDI score	Onset age (years)	Medication (%)	Comorbidity
	AN	HC	AN	HC		AN	HC				
*Samples from adults*											
Amianto et al. (2013)	17(17)	14(14)	20	24	1.08	16.0	21.0	13	NA	Drug naive	No
Bar et al. (2015)	26(23)	26(23)	22.9	24	1.86	16.9	21.7	20	NA	Drug naive	No
Bjornsdotter et al. (2018)	25(25)	25(25)	20.3	21.2	4.14	16.2	21.1	26.88	NA	52	Yes
Boghi et al. (2010)	21(21)	27(27)	29	30.8	11.3	15.5	21.9	NA	NA	100 (SSRI)	NA
Brooks et al. (2011)	14(14)	21(21)	26	26	9.2	15.6	21.4	NA	NA	NA	Yes
Cicerale et al. (2013)	10(10)	8(8)	22	24	1.33	15.9	21.1	NA	20	Drug naive	No
D’Agata et al. (2015)	21(21)	17(17)	21	23	<2	16.1	21.5	NA	NA	Drug naive	No
Fonville et al. (2014)	31(NA)	31(NA)	23	25	7	15.8	21.8	NA	16	39	Yes
Friederich et al. (2011)	12(12)	14(14)	24.3	25.6	6.3	15.9	21.1	NA	NA	Drug naive	No
Joos et al. (2010)	12(12)	18(18)	25	26.9	4.7	16.0	21.2	26.5	NA	8.3	Yes
Kohmura et al. (2017)	23(23)	29(29)	28.5	28.2	10.5	13.2	21.5	25.3	18	NA	No
Phillipou et al. (2018)	26(26)	27(27)	22.8	22.5	6.42	16.6	22.6	NA	16.04	100	Yes
Suchan et al. (2010)	15(15)	15(15)	26.8	29.5	5.5	16.0	22.0	NA	NA	NA	NA
Van Opstal et al. (2015)	10(10)	11(11)	22.1	20.8	3.54	15.6	20.3	NA	NA	25	No
*Samples from adolescents*											
Bomba et al. (2015)	11(11)	8(8)	13.6	13.2	1.20	12.7	19.8	NA	NA	Drug naive	No
Castro Fornieles et al. (2009)	12(11)	9(8)	14.5	14.6	0.69	14.8	NA	NA	NA	25	Yes
Frank et al. (2013)	19(19)	22(22)	15.4	14.8	NA	16.2	21.3	NA	NA	58	Yes
Fujisawa et al. (2015)	20(20)	14(14)	14.1	14.9	1.96	14.3	NA	NA	NA	Drug naive	No
Gaudio et al. (2011)	16(16)	16(16)	15.2	15.1	0.44	14.2	20.2	NA	14.7	100	No
Martin Monzon et al. (2017)	26(26)	20(20)	16.5	17.2	<3	16.6	22.6	NA	NA	NA	No
Olivo et al. (2018)	22(22)	38(38)	14.7	14.8	0.66	19.3	20.7	NA	NA	Drug naive	No

*BMI* body mass index, *AN* anorexia nervosa, *HC* health control, *BDI* Beck Depression Inventory, *R* restrictive subtype of anorexia nervosa, *NA* not available

### Voxel-wise meta-analysis

#### Pooled meta-analyses of all included studies (21 studies)

**Fig. 2:**
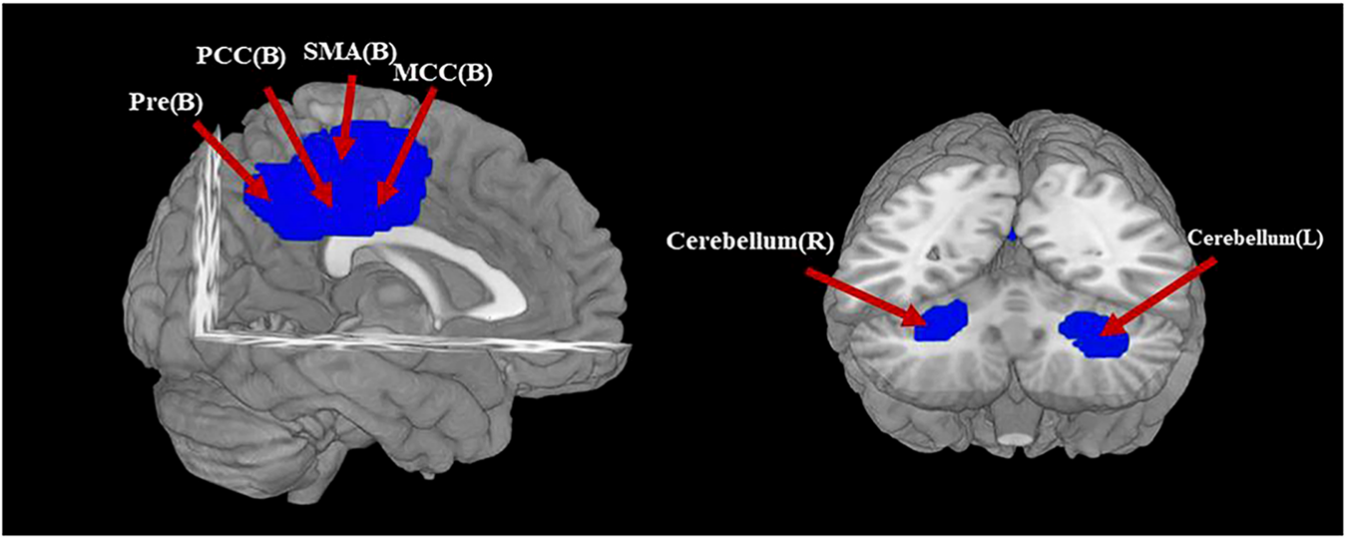
Regions showing reduced gray matter volume in AN patients compared with healthy controls. L left, R right, B bilateral, Pre precuneus, PCC posterior cingulate cortex, SMA supplementary motor area, MCC median cingulate cortex

AN patients showed decreased GMV in the bilateral MCC and PCC, extending to bilateral SMA and precuneus. GMV was also decreased in bilateral cerebellum. No regions were identified with increased GMV in AN (Table 3 and Fig. 2).

**Table 2:** Technique details of the VBM studies on AN included in the meta-analysis

Study	MRI scanner (T)	Software	Smoothing (FWHM) (mm)	*p*-Value	Voxels	Coordinates
Amianto et al. (2013)	1.5	FSLVBM	7	<0.005 (uncorrected)	60	10
Bar et al. (2015)	1.5	SPM8	8	<0.05 (FWE)	NA	5
Boghi et al. (2010)	1.0	SPM2	12	<0.05 (FDR)	NA	19
Bomba et al. (2015)	1.5	SPM5	8	<0.05 (FWE)	NA	5
Brooks et al. (2011)	1.5	SPM5	12	<0.05 (FDR)	NA	6
Bjornsdotter et al. (2018)	3.0	SPM8	8	<0.05 (FWE)	NA	0
Cicerale et al. (2013)	1.5	FSLVBM	7	<0.005 (uncorrected)	60	0
D’Agata et al. (2015)	1.5	FSLVBM	NA	<0.05 (NA)	50	3
Fonville, et al. (2014)	1.5	FSLVBM	7	<0.05 (FWE)	NA	10
Friederich et al. (2011)	3.0	SPM5	8	<0.05 (corrected)	NA	8
Joos et al. (2010)	3.0	SPM8	12	<0.05 (corrected)	NA	7
Kohmura et al. (2017)	3.0	SPM8	8	<0.05 (FWE)	NA	9
Phillipou et al. (2018)	3.0	SPM12	8	<0.05 (FWE)	NA	10
Suchan et al. (2010)	1.5	SPM5	12	< 0.05 (FDR)	NA	2
Van-opstal et al. (2015)	3.0	FSLVBM	NA	<0.05 (NA)	NA	2
CastroFornieles et al. (2009)	1.5	SPM5	12	<0.05 (FWE)	NA	6
Fujisawa et al. (2015)	3.0	SPM8	12	<0.05 (FWE)	NA	2
Gaudio et al. (2011)	1.5	SPM2	8	<0.05 (FWE)	NA	3
MartinMonzon al. (2017)	3.0	SPM12	6	<0.05 (FDR)	NA	25
Frank et al. (2013)	3.0	SPM8	8	<0.05 (FWE)	NA	12
Olivo et al. (2018)	3.0	SPM 12	8	<0.05 (FWE)	NA	0

*FDR* false discovery rate, *FWE* family-wise error correction, *NA* not available, *VBM* voxel-based morphometry

**Table 3:** The regions of decreased gray matter volume in AN patients compared with HC identified by the main meta-analyses

Region	Maximum				Cluster	Jackknife sensitivity analysis
	MNI coordinates *x*, *y*, *z*	SDM *z*-score	*p*-Value uncorrected	Number of voxels	Breakdown (no. of voxels)	
L median cingulate	−4, −30, 44	−3.223	~0	2348	L median cingulate (849) R median cingulate (363) L precuneus (347) R precuneus (269) L supplementary motor area (250) L posterior cingulate gyrus (138) R supplementary motor area (82) R posterior cingulate gyrus (50)	21 of 21
L cerebellum	−28, −54, −32	−2.137	0.001114726	181	L cerebellum, hemispheric lobule VI, BA 37 (96) L cerebellum, hemispheric lobule VI (59) L cerebellum, hemispheric lobule VI, BA 19 (18) L cerebellum, crus I (8)	18 of 21 (Amianto et al.; D’Agata et al.; Fonville et al.)
R cerebellum, crus I	30, −50, −22	−2.011	0.001207650	85	R cerebellum, crus I (42) R cerebellum, hemispheric lobule VI (43)	19 of 21 (Fonville et al.; Phillipou et al.)

The jackknife sensitivity analysis column gives the number of studies whose omission does not affect the finding, and abbreviated reference citations for the remainder which do

*BA* Brodmann area, *GM* gray matter, *MNI* Montreal Neurological Institute Space, *L* left, *R* right, *SDM* seed-based d mapping

#### Subgroup meta-analyses of adult AN studies (14 studies)

**Fig. 3:**
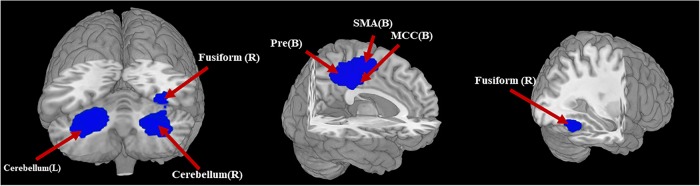
Regions showing reduced gray matter volume in AN-adult patients compared with healthy controls. L left, R right, B bilateral, Pre precuneus, SMA supplementary motor area, MCC median cingulate cortex

The adult AN subgroup showed decreased GMV in bilateral MCC and left PCC, extending to bilateral SMA and precuneus. GMV was also decreased in right fusiform gyrus and bilateral cerebellum. No regions were identified with increased GMV in AN (Table 4 and Fig. 3).

**Table 4:** Regional differences in gray matter volume in AN patients compare with HC subjects identified in the two subgroup meta-analyses

Region	Maximum				Cluster breakdowns	Jackknife sensitivity analysis
	MNI coordinates *x*, *y*, *z*	SDM *z*-score	*p*-Value uncorrected	Number of voxels		
*Samples from AN adults (14 datasets)*						
Decreased GMV (AN < HC)						
R fusiform gyrus	32, −56, −18	−2.113	0.001207650	137	R fusiform (137)	12 of 14 (D’Agata et al.; Fonville et al.)
R cerebellum	30, −52, −22	−2.045	0.001744330	433	R cerebellum (433)	14 of 14
L cerebellum,	−24, −54, −28	−2.432	0.000108361	898	L cerebellum (733)	14 of 14
L median cingulate	−4, −30, 46	−2.455	0.000098050	839	L median cingulate (415) R median cingulate (84) L supplementary motor area (155) R supplementary motor area (60) R precuneus (36) L precuneus (82) L posterior cingulate gyrus (7)	14 of 14
*Samples from pure AN patients (12 datasets)*						
Decreased GMV (AN < HC)						
L supplementary motor area	−4, −18, 52	−2.784	~0	1841	L median cingulate (660) R median cingulate (422) L supplementary motor area (335) R supplementary motor area (189) L posterior cingulate gyrus (94) R posterior cingulate gyrus (41) R precuneus (82) L precuneus (18)	12 of 12
L amygdala	−30, −4, −20	−1.773	0.002250135	130	L amygdala (130)	9 of 12 (Amianto et al., Friederich et al, MartinMonzon et al.)
L anterior cingulate	2, 38, 24	−1.746	0.002699077	43	L anterior cingulate (43)	9 of 12 (Kohmura et al., MartinMonzon et al., Van-opstal et al.)

The jackknife sensitivity analysis column gives the number of studies whose omission does not affect the finding, and abbreviated reference citations for the remainder which do

*BA* Brodmann area, *GM* gray matter, *MNI* Montreal Neurological Institute Space, *L* left, *R* right, *SDM* seed-based d mapping

#### Subgroup meta-analyses of pure AN studies (12 studies)

**Fig. 4:**
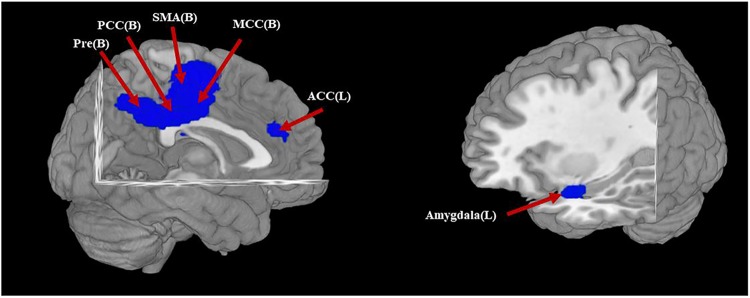
Regions showing reduced gray matter volume in pure (non-comorbid) AN patients compared with healthy controls. L left, R right, B bilateral, Pre precuneus, PCC posterior cingulate cortex, SMA supplementary motor area, MCC median cingulate cortex, ACC anterior cingulate cortex

The pure AN patient subgroup showed decreased GMV in bilateral MCC and PCC extending to bilateral SMA and precuneus. GMV was also decreased in the left amygdala and left ACC (Table 4 and Fig. 4).

### Jackknife sensitivity analysis

The main findings remained largely unchanged in jackknife sensitivity analysis: the detailed results are provided in Tables 3 and 4.

### Meta-regression analysis

We examined the potential effect of relevant clinical variables by means of simple linear regression using SDM. The mean age, percentage of female patients, BMI, illness duration, and percentage of medicated patients were not linearly associated with GMV changes. Limited data precluded meta-regression analysis of structural change for the Beck Depression Inventory (BDI) score. We were unable to assess the relationship to AN symptom severity, because this was reported using a variety of incompatible measures.

## Discussion

The present study is an up-to-date meta-analysis using the powerful technique of SDM to define the differences of GMV between AN patients and HCs, based on 21 VBM studies of 389 AN and 410 HC. There are three robust findings. First, the whole group of AN patients showed decreased GMV in bilateral MCC, PCC extending to the bilateral SMA, precuneus, and cerebellum. Second, GMV was decreased in the right fusiform gyrus only in adult AN. Third, GMV was decreased in the left amygdala and left ACC only in pure AN. Overall, the cingulate cortex, the frontal, and parietal lobes seem to be especially involved in AN.

### Implications of findings in AN patients as a whole

As hypothesized, the results robustly demonstrated significantly decreased GMV in the cingulate cortex. We did not find differences in areas primarily underlying reward processing, such as the striatum, often reported as most susceptible to decreased GMV^10,33,34^, this difference may be due to our larger number of studies and more accurate methodology. The key regions showing decreased GMV were the bilateral MCC, PCC extending to the bilateral precuneus, and SMA. These results accord with previous structural neuroimaging findings^6,35^. What might they mean?

The MCC is involved in identifying the emotional significance of a stimulus to produce an appropriate affective state and behavioral response; the anterior sub-region of MCC seems to be particularly involved in fear and avoidance behavior, and it has greater amygdala input than other cingulate regions^36^. AN features disturbance in both emotional and cognitive function, and the involvement of MCC could explain much of the specific symptomatology, such as inhibition, anxiety, depression, and alexithymia.

The precuneus and the PCC are key components of the default mode network (DMN), which is mainly involved in self-reflection^37^. Studies using task-based fMRI have shown reduced DMN activity in women with AN^38,39^. Thus, the alteration in DMN may point to an abnormality of the regulation of subjectivity and conscious self-monitoring^40,41^, perhaps underlying these patients’ rigid cognitive strategies to control food intake, and lack of recognition of starvation.

SMA volumetric decrease has previously been reported in AN^18,19^. The SMA is related to the planning and control of motor actions, and plays a pivotal role in task switching, particularly in proactive behavioral switching^42,43^. An impairment in this region may contribute to the patients’ cognitive-behavioral inflexibility^44^, which may underlie their self-induced starvation. Furthermore, a task-related fMRI study of female AN patients also identified reduced sensorimotor network (SMN) activity in the SMA^39^; this may imply that SMN impairments in AN reflect dysfunctional processing of somatosensory information regarding body size. A decrease of activation in this area may therefore facilitate the body dissatisfaction which is a core symptom of AN.

Our finding of decreased cerebellum GMV is consistent with previous studies^18,21,22,45^, and a recent resting state fMRI study demonstrated altered intrinsic connectivity of the cerebellar vermis in AN patients^46^. There is accumulating evidence that the cerebellum is involved in the regulation of various visceral functions including feeding control^47,48^. Patients with AN present both misperception of visceral feedback, such as feeling of fullness, and an inflexible cognitive pattern that prevents them from modifying their behavior. We hypothesize that GMV changes and dysfunctional neural patterns in the cerebellum might contribute to core symptoms of AN, such as self-induced starvation and food aversion.

### Implications of findings in the adult AN subgroup

A noteworthy finding is decreased GMV in the right fusiform gyrus in adult AN. The fusiform gyrus is involved in body size perception and food processing^49^, and abnormalities in it could underlie AN patients’ impaired perception of their own body, as well as their cognitive bias in food imaging and processing^21,50^. There is also evidence of reduced effective connectivity between the left fusiform body area and the extrastriate body area in AN^51,52^. However, because it was impossible to perform meta-analysis of the adolescent AN group, it remains unclear whether the fusiform area is more vulnerable in adult AN than in adolescent AN.

### Implications of findings in the pure AN subgroup

Nearly three-quarters of AN patients report a lifetime mood disorder, such as MDD, anxiety disorder or OCD^1^. Investigating the pure AN patient subgroup therefore offers the best opportunity to gain evidence for neural pathology directly associated with the disease. Interestingly, the pure AN subgroup demonstrated decreased GMV in the left amygdala and the left ACC, confirming previous studies^53,54^. The amygdala is involved in the expression of fear and anxiety, and also influences emotional processes, such as emotional learning and emotional regulation^55,56^. Furthermore, previous fMRI research has revealed hyperactivation of the amygdala in AN patients in response to looking at their own body image^57^. Thus, our findings suggest that morphometric alterations in amygdala may underlie an intense fear of weight gain in AN.

The ACC is involved in reward networks^58^ and affective processing^59^. It could also be related to the deficit in set-shifting which is a neuropsychological trait in AN^58^. In a functional MRI study of this, AN patients showed less activity in the ACC^60^.

However, we did not observe any GMV alteration in amygdala and ACC in the pooled whole-group results. The reason for this discrepancy is unclear, although it could be that changes associated with comorbidity, such as MDD, anxiety disorder, and OCD may normalize, or at least obscure, the intrinsic changes in these sensitive regions.

### Implications of non-significant findings in meta-regression analysis

Although no significant correlations were found between clinical variables and GM changes, some potential factors may impact on GMV, among which illness duration and BMI were of particular interest to us.

Three studies demonstrated that illness duration was related to GM volume changes^16,17,21^, however, this was not confirmed in other studies^10,12,18,20,22,61^. These inconsistent results can be read in two ways: (1) because of heterogeneous patient groups with respect to AN subtype ratio, presence of medication, and comorbidity in the included studies, it may be that our meta-regression lacked sufficient power to detect any such effect; (2) GM changes in AN patients might emerge before the onset and continue in the same way, regardless of the duration^12^.

Previous VBM studies reported either significant correlations^10,18,21,22,11^ or no correlations^6,16,17,20,62^ between BMI and GMV in different brain regions. It is possible that these divergent findings may simply have canceled out in our meta-analysis. Alternatively, morphological impairments might best be considered not as a direct consequence of malnutrition, but rather as a premorbid symptom of AN that accompanies neuropsychological impairments^16^. However, these preliminary results need confirmation in more longitudinal studies.

### Limitations

Our study has several limitations. First, like most voxel-wise meta-analyses, it was based on the published coordinates rather than raw statistical brain maps, which may result in less accurate results^30^. Second, we could not take AN-subtypes into account. The restricting subtype and the binge-purging subtype may have different etiologies, but this was impossible to investigate because the information was not available in the included studies. Third, some of patients in the meta-analysis were taking antidepressant medication, which may itself affect brain structure^63^. Finally, although we found gray matter changes which were different from the pooled results in adult AN and pure AN, it cannot be concluded that these changes are characteristic of these subgroups, because the changes in their comparative groups (adolescent AN and AN with comorbidity) are still unknown. More studies on these subgroups are needed to reach the minimum requirement for reliable meta-analysis.

## Conclusion

The present results robustly suggest that patients with AN have significantly decreased GMV in brain regions which are involved in DMN and SMN. These structural abnormalities are consistent with previously reported functional changes, and may therefore underpin the pathophysiological alternations and thus offer some explanation of the core symptomology of AN. Future longitudinal studies in at-risk populations are needed to validate these findings and to clarify whether the observed changes are the cause or the consequence of this illness. This may help development of strategies that strengthen resilience, as well as treatments to normalize these alterations.

## References

[1] Zipfel S, Giel KE, Bulik CM, Hay P, Schmidt U (2015). Anorexia nervosa: aetiology, assessment, and treatment. Lancet Psychiatry.

[2] Nielsen S (2001). Epidemiology and mortality of eating disorders. Psychiatr. Clin. North Am..

[3] Zipfel S (2013). Impact of exercise on energy metabolism in anorexia nervosa. J. Eat. Disord..

[4] Treasure J, Claudino AM, Zucker N (2008). Eating disorders. Lancet.

[5] Bakalar JL, Shank LM, Vannucci A, Radin RM, Tanofsky-Kraff M (2015). Recent advances in developmental and risk factor research on eating disorders. Curr. Psychiatry Rep..

[6] Joos A (2010). Voxel-based morphometry in eating disorders: correlation of psychopathology with grey matter volume. Psychiatry Res..

[7] Suchan B (2010). Reduction of gray matter density in the extrastriate body area in women with anorexia nervosa. Behav. Brain Res..

[8] Titova OE, Hjorth OC, Schioth HB, Brooks SJ (2013). Anorexia nervosa is linked to reduced brain structure in reward and somatosensory regions: a meta-analysis of VBM studies. BMC Psychiatry.

[9] Seitz J (2014). Morphological changes in the brain of acutely ill and weight-recovered patients with anorexia nervosa. A meta-analysis and qualitative review. Z. Kinder Jugend. Psychother..

[10] Phillipou A (2018). Differences in regional grey matter volumes in currently ill patients with anorexia nervosa. Eur. J. Neurosci..

[11] Martin Monzon B (2017). Grey matter volume in adolescents with anorexia nervosa and associated eating disorder symptoms. Eur. J. Neurosci..

[12] Kohmura K (2017). Regional decrease in gray matter volume is related to body dissatisfaction in anorexia nervosa. Psychiatry Res..

[13] Fujisawa TX (2015). Anorexia nervosa during adolescence is associated with decreased gray matter volume in the inferior frontal gyrus. PLoS ONE.

[14] D'Agata F (2015). Brain correlates of alexithymia in eating disorders: a voxel-based morphometry study. Psychiatry Clin. Neurosci..

[15] Bomba M (2015). Global and regional brain volumes normalization in weight-recovered adolescents with anorexia nervosa: preliminary findings of a longitudinal voxel-based morphometry study. Neuropsychiatr. Dis. Treat..

[16] Bar KJ, de la Cruz F, Berger S, Schultz CC, Wagner G (2015). Structural and functional differences in the cingulate cortex relate to disease severity in anorexia nervosa. J. Psychiatry Neurosci..

[17] Fonville L, Giampietro V, Williams SC, Simmons A, Tchanturia K (2014). Alterations in brain structure in adults with anorexia nervosa and the impact of illness duration. Psychol. Med..

[18] Amianto F (2013). Brain volumetric abnormalities in patients with anorexia and bulimia nervosa: a voxel-based morphometry study. Psychiatry Res..

[19] Friederich HC (2012). Grey matter abnormalities within cortico-limbic-striatal circuits in acute and weight-restored anorexia nervosa patients. Neuroimage.

[20] c S (2011). Gray matter decrease distribution in the early stages of Anorexia Nervosa restrictive type in adolescents. Psychiatry Res.: Neuroimaging.

[21] Brooks SJ (2011). Restraint of appetite and reduced regional brain volumes in anorexia nervosa: a voxel-based morphometric study. BMC Psychiatry.

[22] Boghi A (2011). In vivo evidence of global and focal brain alterations in anorexia nervosa. Psychiatry Res..

[23] Castro-Fornieles J (2009). A cross-sectional and follow-up voxel-based morphometric MRI study in adolescent anorexia nervosa. J. Psychiatr. Res..

[24] Frank GKW, Shott ME, Hagman JO, Yang TT (2013). Localized brain volume and white matter integrity alterations in adolescent anorexia nervosa. J. Am. Acad. Child Adolesc. Psychiatry.

[25] van Opstal AM (2015). Hypothalamic BOLD response to glucose intake and hypothalamic volume are similar in anorexia nervosa and healthy control subjects. Front. Neurosci..

[26] Liberati A (2009). The PRISMA statement for reporting systematic reviews and meta-analyses of studies that evaluate healthcare interventions: explanation and elaboration. Br. Med. J. (Clin. Res. Ed.).

[27] Lim L, Radua J, Rubia K (2014). Gray matter abnormalities in childhood maltreatment: a voxel-wise meta-analysis. Am. J. Psychiatry.

[28] Hu X (2017). Meta-analytic investigations of common and distinct grey matter alterations in youths and adults with obsessive-compulsive disorder. Neurosci. Biobehav. Rev..

[29] Wang W (2017). Conjoint and dissociated structural and functional abnormalities in first-episode drug-naive patients with major depressive disorder: a multimodal meta-analysis. Sci. Rep..

[30] Radua J (2012). A new meta-analytic method for neuroimaging studies that combines reported peak coordinates and statistical parametric maps. Eur. Psychiatry.

[31] Radua J (2014). Anisotropic kernels for coordinate-based meta-analyses of neuroimaging studies. Front. Psychiatry.

[32] Carlisi CO (2017). Comparative multimodal meta-analysis of structural and functional brain abnormalities in autism spectrum disorder and obsessive-compulsive disorder. Biol. Psychiatry.

[33] Favaro A, Tenconi E, Degortes D, Manara R, Santonastaso P (2014). Effects of obstetric complications on volume and functional connectivity of striatum in anorexia nervosa patients. Int. J. Eat. Disord..

[34] Frank GK (2013). Altered brain reward circuits in eating disorders: chicken or egg?. Curr. Psychiatry Rep..

[35] Gaudio S (2011). Gray matter decrease distribution in the early stages of anorexia nervosa restrictive type in adolescents. Psychiatry Res..

[36] Vogt BA, Finch DM, Olson CR (1992). Functional heterogeneity in cingulate cortex: the anterior executive and posterior evaluative regions. Cereb. Cortex (New York, NY: 1991).

[37] Buckner RL, Andrews-Hanna JR, Schacter DL (2008). The brain's default network: anatomy, function, and relevance to disease. Ann. N. Y. Acad. Sci..

[38] Sachdev P, Mondraty N, Wen W, Gulliford K (2008). Brains of anorexia nervosa patients process self-images differently from non-self-images: an fMRI study. Neuropsychologia.

[39] Mcfadden KL, Tregellas JR, Shott ME, Frank GK (2014). Reduced salience and default mode network activity in women with anorexia nervosa. J. Psychiatr. Neurosci. Jpn..

[40] Lou HC (2004). Parietal cortex and representation of the mental self. Proc. Natl Acad. Sci. USA.

[41] Wagner G (2013). Self-referential processing influences functional activation during cognitive control: an fMRI study. Soc. Cogn. Affect. Neurosci..

[42] Nachev P, Kennard C, Husain M (2008). Functional role of the supplementary and pre-supplementary motor areas. Nat. Rev. Neurosci..

[43] Hikosaka O, Isoda M (2010). Switching from automatic to controlled behavior: cortico-basal ganglia mechanisms. Trends Cogn. Sci..

[44] Friederich HC, Herzog W (2011). Cognitive-behavioral flexibility in anorexia nervosa. Curr. Top. Behav. Neurosci..

[45] Husain MM (1992). Subcortical brain anatomy in anorexia and bulimia. Biol. Psychiatry.

[46] Amianto F (2013). Intrinsic connectivity networks within cerebellum and beyond in eating disorders. Cerebellum.

[47] Mahler P, Guastavino JM, Jacquart G, Strazielle C (1993). An unexpected role of the cerebellum: involvement in nutritional organization. Physiol. Behav..

[48] Zhu JN, Wang JJ (2008). The cerebellum in feeding control: possible function and mechanism. Cell. Mol. Neurobiol..

[49] Downing PE, Jiang Y, Shuman M, Kanwisher N (2001). A cortical area selective for visual processing of the human body. Science.

[50] Hummel D (2013). Neural adaptation to thin and fat bodies in the fusiform body area and middle occipital gyrus: an fMRI adaptation study. Hum. Brain Mapp..

[51] Ehrlich S (2015). Reduced functional connectivity in the thalamo-insular subnetwork in patients with acute anorexia nervosa. Hum. Brain Mapp..

[52] Suchan B (2013). Reduced connectivity between the left fusiform body area and the extrastriate body area in anorexia nervosa is associated with body image distortion. Behav. Brain Res..

[53] Giordano GD (2001). Volume measurement with magnetic resonance imaging of hippocampus-amygdala formation in patients with anorexia nervosa. J. Endocrinol. Invest..

[54] McCormick LM (2008). Implications of starvation-induced change in right dorsal anterior cingulate volume in anorexia nervosa. Int. J. Eat. Disord..

[55] Phelps EA, LeDoux JE (2005). Contributions of the amygdala to emotion processing: from animal models to human behavior. Neuron.

[56] Davis M, Whalen PJ (2001). The amygdala: vigilance and emotion. Mol. Psychiatry.

[57] Joos AAB (2011). Amygdala hyperreactivity in restrictive anorexia nervosa. Psychiatr. Res.: Neuroimaging.

[58] Holliday J, Tchanturia K, Landau S, Collier D, Treasure J (2005). Is impaired set-shifting an endophenotype of anorexia nervosa?. Am. J. Psychiatry.

[59] Bush G, Luu P, Posner MI (2000). Cognitive and emotional influences in anterior cingulate cortex. Trends Cogn. Sci..

[60] Zastrow A (2009). Neural correlates of impaired cognitive-behavioral flexibility in anorexia nervosa. Am. J. Psychiatry.

[61] Olivo G (2018). Atypical anorexia nervosa is not related to brain structural changes in newly diagnosed adolescent patients. Int. J. Eat. Disord..

[62] Bjornsdotter M (2018). Grey matter correlates of autistic traits in women with anorexia nervosa. J. Psychiatr. Neurosci..

[63] Lavretsky H, Roybal DJ, Ballmaier M, Toga AW, Kumar A (2005). Antidepressant exposure may protect against decrement in frontal gray matter volumes in geriatric depression. J. Clin. Psychiatry.

[64] Kressel, H. Y. Setting Sail: 2017. *Radiology* **282**, 4–6 (2017). 10.1148/radiol.2016162471 28005504

[65] Lui, S., Zhou, X. J., Sweeney, J. A., & Gong, Q. Psychoradiology: The Frontier of Neuroimaging in Psychiatry. *Radiology* **281**, 357–372 (2016). 10.1148/radiol.2016152149 PMC5084981 27755933

[66] Port, J. D. Diagnosis of Attention Deficit Hyperactivity Disorder by Using MR Imaging and Radiomics: A Potential Tool for Clinicians. *Radiology* **287**,631–632(2018). 10.1148/radiol.2018172804 29668406

[67] Sun, H. et al. Psychoradiologic Utility of MR Imaging for Diagnosis of Attention Deficit Hyperactivity Disorder: A Radiomics Analysis. *Radiology* **287**, 620–630 (2018). 10.1148/radiol.2017170226 29165048

